# COVID-19 Lockdowns and Hospitalisations for Oro-Facial Trauma Among Adults in Australia and the United Kingdom

**DOI:** 10.3390/healthcare13070789

**Published:** 2025-04-01

**Authors:** Parmis Aminian, Marc Tennant, Estie Kruger

**Affiliations:** School of Allied Health, The University of Western Australia, Crawley 6009, Australia; marc.tennant@uwa.edu.au (M.T.); estie.kruger@uwa.edu.au (E.K.)

**Keywords:** hospitalisation, lockdown, oro-facial, trauma, Australia, UK, adult

## Abstract

**Objectives:** Oro-facial trauma affects physical, psychological, and social well-being. This study assesses changes in oro-facial trauma hospitalisation rates among adults during the pre-lockdown period, ‘lockdown year’, and post-lockdown period in Australia and the United Kingdom (UK). The findings provide insights into healthcare delivery and resource allocation during public health emergencies to inform future preventive strategies. **Methods:** Hospitalisation data for adults (aged 20+) with oro-facial trauma, classified using ICD-10 codes, were collected. Age-standardised rates (ASRs) per 100,000 population were calculated for each period. Comparative analyses evaluated hospitalisation rates during the ‘lockdown year’ relative to three years before and after. The 11 oro-facial trauma ICD codes were grouped into major and minor trauma to evaluate differential impacts. **Results:** This study highlighted a statistically significant reduction in oro-facial trauma hospitalisation rates during the ‘lockdown year’ in both Australia (38.8%) and the UK (35.7%) compared to the pre-lockdown period. Although rates increased post-lockdown, they remained lower than pre-lockdown levels, with a 35.5% reduction in Australia and a 25.1% reduction in the UK. Additionally, while the ASR significantly increased for major trauma in the post-lockdown years compared to the lockdown year, the increase for minor trauma was not statistically significant in both countries. **Conclusions:** COVID-19 lockdowns led to a significant reduction in oro-facial trauma hospitalisations. Post-lockdown rates increased as activities resumed but did not return to pre-lockdown levels, suggesting lasting behavioural shifts. These findings highlight the role of external factors (e.g., mobility and social behaviour) in oro-facial trauma rates and can inform targeted preventive strategies for high-risk periods.

## 1. Introduction

Oro-facial trauma is a notable health concern that is often accidental and challenging to prevent entirely. These injuries commonly burden emergency departments and result in hospitalisations. Understanding the epidemiology, causes, risk factors, and impact of a situation may help develop strategies to reduce hospitalisation rates. This knowledge can aid in designing management approaches to mitigate the impact on the healthcare system, both in terms of resources and costs. Previous studies have identified factors associated with oro-facial trauma, including alcohol consumption, non-compliance with road traffic laws, interpersonal violence, sports and travel accidents, work-related accidents, falls, and conditions like osteoporosis [1,2].

Coronavirus disease 2019 (COVID-19), caused by the SARS-CoV-2 virus, emerged in late 2019 in Wuhan, China, and rapidly spread worldwide [3]. To prevent its transmission, lockdowns were implemented, along with restrictions. The pandemic disrupted oral health services. Many nonessential surgeries in hospitals were postponed worldwide to minimise the spread of the disease and conserve resources [4,5,6]. Lockdowns of workplaces, universities, sports centres, pubs, clubs, and restrictions on social gatherings and travel during the pandemic influenced the incidence of oro-facial trauma. This study aims to assess changes in oro-facial trauma hospitalisation rates among adults during the pre-lockdown period, ‘lockdown year’, and post-lockdown period at a national level in Australia and the UK. Identifying these changes could help develop protocols to improve hospital preparedness in similar future scenarios.

## 2. Materials and Methods

This study was a retrospective, descriptive analysis. Data were collected for all adults aged 20 years and older in Australia and the UK, focusing on hospitalisations (defined as admissions with completed episodes of care) related to oro-facial trauma. The anonymous dataset included year, age, and corresponding International Classification of Disease, 10th Revision (ICD-10) codes for conditions identified as primary diagnoses of oro-facial trauma, specifically those relevant to dentistry. Hospitalisation data were standardised by using ICD-10 codes. Eleven ICD-10 codes were included: S00.5 (superficial injury of lip and oral cavity), S01.5 (open wound of lip and oral cavity), S02.2 (fracture of nasal bone), S02.3 (fracture of orbital floor), S02.4 (fracture malar and maxillary bones), S02.5 (fracture of tooth), S02.6 (fracture of mandible), S03.0 (dislocation of jaw), S03.1 (dislocation of septal cartilage of nose), S03.2 (dislocation of tooth), S03.4 (sprain and strain of jaw).

As part of this study, the 11 oro-facial trauma ICD codes were divided into two groups: major traumas and minor traumas. In this study, major traumas include S02.2 (fracture of nasal bone), S02.3 (fracture of orbital floor), S02.4 (fracture of malar and maxillary bones), S02.6 (fracture of mandible), and S03.1 (dislocation of septal cartilage of the nose), which are generally considered to require specialised management and tertiary care or hospital admission due to the complexity and severity of the conditions, and minor traumas include S00.5 (superficial injury of lip and oral cavity), S01.5 (open wound of lip and oral cavity), S02.5 (fracture of tooth), S03.0 (dislocation of jaw), S03.2 (dislocation of tooth), and S03.4 (sprain and strain of jaw), representing less severe conditions that may be managed in primary or secondary care settings without the need for tertiary-level interventions. This classification aimed to examine which group of trauma conditions was more affected following the lockdowns.

Australian data were sourced from open-access datasets provided by the Australian Institute of Health and Welfare (AIHW) [7]. The UK data were obtained from the National Health Service (NHS) website [8]. Population data for age groups were retrieved from the Australian Bureau of Statistics (ABS) for Australian populations and from the Office for National Statistics (ONS) government website for UK populations. All results were reported according to the financial year [9,10].

Age-standardised rates (ASRs) per 100,000 population were calculated for each age group. Data analysis was conducted using Microsoft Excel (version 16.66.1). Differences between rates, along with 95% confidence intervals (CIs) and associated *p*-values, were calculated for three years before the lockdown year (pre-lockdown period), the ‘lockdown year’ itself, and three years following the lockdown year (post-lockdown period). A *p*-value of less than 0.05 was considered statistical significance. Chi-squared tests were used to compare hospitalisation rates during the ‘lockdown year’ with the average rates in the preceding three years and the subsequent three years [11].

Due to slight differences in the financial year dates between Australia and the UK, as well as variations in the timing of lockdown times, the majority of lockdowns occurred during the 2019–20 financial year in Australia, whereas in the UK, the majority of lockdowns fell within the 2020–21 financial year. Therefore, in this study, the 2019–20 financial year was designated as the ‘lockdown year’ for Australia, while the 2020–21 financial year was considered the ‘lockdown year’ for the UK [12,13,14].

In this study, the ‘pre-lockdown period’ refers to the average range of data from the three years preceding the ‘lockdown year’, while the ‘post-lockdown period’ refers to the average range of data from the three years following the ‘lockdown year’. In addition, adults aged 20–39 were categorised as ‘younger’, those aged 40–59 as ‘middle-aged’, and those aged 60 and above as ‘elderly’.

## 3. Results

### 3.1. Australia

Over the study period from 2016–17 to 2022–23, the highest number of oro-facial trauma hospitalisations among Australian adults occurred in younger males, primarily due to major trauma conditions (Table 1). Among males, those aged 20–24 years had the highest rates of oro-facial hospitalisations, while among females, those aged 85 years and older consistently had the highest rates each year throughout the study period.

**Figure 1:**
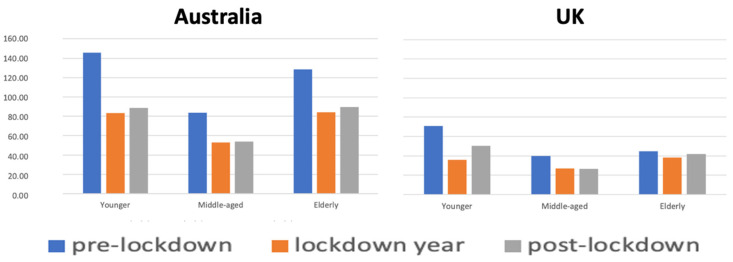
The ASR of oro-facial trauma hospitalisations among adults in Australia and the UK, comparing pre-lockdown, ‘lockdown year’, post-lockdown by age groups. pre-lockdown: the average of ASR hospitalisations during the three years preceding the lockdown year; ‘lockdown year’: in Australia (2019–20) and in UK (2020–21); post-lockdown: the average of ASR hospitalisations during the three years following the lockdown year. Younger: 20–39 years old; Middle-aged: 40–59 years old; Elderly: 60^+^ years old. ASR calculated by 100,000 population of the age group.

The age-standardised rate (ASR) of oro-facial trauma hospitalisations for adults aged over 20 years was 120.07 per 100,000 population during the pre-lockdown period. This rate decreased significantly to 73.51 during the ‘lockdown year’ and then slightly increased to 77.39 in the post-lockdown period, though it remained below the pre-lockdown level (Table 2). These changes were consistent across different age groups, including younger, middle-aged, and elderly individuals (Table 2). The reduction in ASR oro-facial trauma hospitalisations during the ‘lockdown year’ was statistically significant for all age groups (*p* < 0.05). The increase in hospitalisations during the post-lockdown period compared to the ‘lockdown year’ was significant for all age groups (*p* < 0.05), except the middle-aged group (*p* = 0.49). The analysis revealed a statistically significant reduction in oro-facial trauma hospitalisation rates during the post-lockdown period compared to the pre-lockdown period across all age groups (*p* < 0.05) (Figure 1).

This study found that oro-facial trauma hospitalisation rates among Australian adults aged 20 and over decreased by 38.8% during the ‘lockdown year’ compared to the pre-lockdown period. While the rates increased after the lockdown year, they remained 35.5% lower in the post-lockdown period than in the pre-lockdown period.

#### Major Trauma vs. Minor Trauma in Australia

The number of hospitalisations for major trauma conditions exceeded those for minor trauma conditions across all age groups during the pre-lockdown period, the ‘lockdown year’, and the post-lockdown period (Table 1).

**Figure 2:**
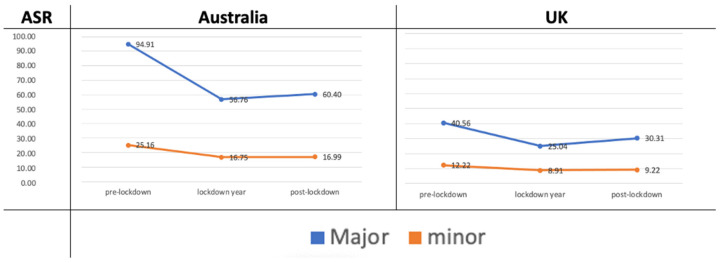
ASR oro-facial trauma hospitalisations (Major vs. minor trauma conditions) among adults (age 20+) during the pre-lockdown period, lockdown year, and post-lockdown period in Australia and the UK.

This study revealed significant reductions in the ASR of hospitalisations for both major and minor trauma among adults aged 20+ during the ‘lockdown year’ compared to the pre-lockdown period. Similarly, a significant reduction was observed in the post-lockdown period compared to the pre-lockdown period for both trauma types (*p* < 0.0001). However, while the increase in ASR during the post-lockdown period compared to the ‘lockdown year’ was significant for major trauma, this increase was not significant for minor trauma (*p* = 0.56) (Table 2, Figure 2).

The incidence rate ratio (IRR) of major traumas to minor traumas was 3.8 during the pre-lockdown period; it decreased in ‘lockdown year’ (3.4) and partially rebounded during the post-lockdown period (3.6), indicating that major trauma consistently occurred more frequently than minor trauma. The age-standardised rate (ASR) for major traumas decreased by 40.2% (from 94.91 to 56.76), while minor trauma showed a 33.4% reduction (from 25.16 to 16.75) in the ‘lockdown year’ (Table 2). Although major trauma had a larger absolute reduction (38.15 compared to 8.41), the proportional decline was greater for minor trauma, leading to a decrease in the IRR for major to minor trauma during the ‘lockdown year’.

Comparing the post-lockdown period to the pre-lockdown period in Australia showed that major trauma rates dropped by 36% and minor trauma rates by 32%, with major trauma experiencing a slightly greater reduction.

### 3.2. UK

This study found that the average annual number of oro-facial hospitalisations for adults aged 20 and above in the UK was 25,156 during the three years prior to the lockdown. This number dropped significantly to 16,409 in the ‘lockdown year’ and then rose to 20,610 in the post-lockdown period.

The ASR showed a significant reduction across all age groups during the ‘lockdown year’. While the ASR increased slightly after the ‘lockdown year’, the rise was not statistically significant for middle-aged adults (40–59 years). However, there was a statistically significant reduction in ASR for all adult age groups in the post-lockdown period compared to the pre-lockdown period (Figure 1).

The analysis showed that oro-facial trauma hospitalisation rates among the UK adults aged 20 and over decreased by 35.7% during the ‘lockdown year’ compared to the pre-lockdown period. Although the rates increased after the lockdown year, they remained 25.1% lower in the post-lockdown period compared to the pre-lockdown period.

#### Major Trauma vs. Minor Trauma in the UK

For adults aged 20 and above, the ASR of hospitalisations showed a significant reduction in both major and minor oro-facial trauma during the ‘lockdown year’ compared to the pre-lockdown period (*p* < 0.0001). Similarly, the ASRs of hospitalisations for both major and minor traumas significantly decreased in the post-lockdown period compared to the pre-lockdown period (*p* < 0.0001). Major trauma hospitalisation rates significantly increased in the post-lockdown period compared to the ‘lockdown year’ (*p* < 0.0001), while the rise in minor trauma hospitalisations during the same period was not statistically significant (*p* = 0.0980) (Figure 2).

In the UK, the incidence rate ratio (IRR) of major to minor trauma was 3.3 during the pre-lockdown period, decreased to 2.8 during the ‘lockdown year’, and returned to 3.3 in the post-lockdown period.

## 4. Discussion

The decrease in oro-facial trauma hospitalisations during the ‘lockdown year’ was statistically significant across all adult age groups in Australia and the UK. Similar findings have been reported in other countries, where reduced physical activities, travel bans, and restrictions on social gatherings during lockdowns lowered the risk factors for trauma incidents [15]. Elderly individuals, who are at the highest risk for falls and related facial fractures [16], experienced a reduced risk as they spent less time outdoors during lockdowns [17]. Additionally, to limit virus transmission and manage hospital staff shortages, hospital admissions were prioritised during this period [18].

Australia’s IRR trends indicate that while both major and minor oro-facial trauma hospitalisations declined during lockdowns, the reduction in major trauma was relatively smaller. This could be due to major trauma incidents being less preventable or because restrictions, such as reduced travel and social activities, primarily impacted preventable minor trauma cases. Additionally, hospitals may have prioritised severe cases, leading to fewer hospitalisations for minor trauma despite ongoing incidents. Changes in daily activities, such as reduced recreational and workplace injuries, likely contributed to the disproportionate reduction in minor trauma. The partial rebound in IRR post-lockdown suggests that as restrictions eased, the relative incidence of major trauma increased, possibly due to a return to higher-risk activities.

The IRR of major traumas to minor traumas was higher in the pre-lockdown period, 3.8, compared to IRR in the post-lockdown period (3.6). This indicates that major trauma was consistently more common than minor trauma during both periods. Hence, major trauma contributes a significantly larger proportion of the total oro-facial trauma burden compared to minor trauma, regardless of the time frame.

Since the differences were statistically significant, these findings are unlikely to be due to random chance and reflect true differences in the incidence of major and minor trauma before and after the ‘lockdown year’. A difference in how the pandemic affected the occurrence of major versus minor trauma may be due to changes in social behaviour or healthcare accessibility.

Several studies conducted in various countries have documented a decline in trauma cases, emergency presentations, or trauma surgeries during the COVID-19 pandemic [4,5,6]. However, this study specifically focused on oro-facial trauma at a national level in two countries over the same study period, encompassing three years before the lockdown year, the ‘lockdown year’, and three years after the lockdown. It analysed the same adult age groups and specific ICD-coded conditions in two countries. The inclusion of age-standardised rates further enhanced the reliability of the findings. On the other hand, some differences in data collection and context between Australia and the UK posed challenges for direct comparison. Australian data included both public and private hospitals, whereas the UK data excluded privately paid hospitalisations. Additionally, the duration and stringency of lockdown measures varied between the two countries, and the financial year calendars differed—Australia’s runs from 1 July to 30 June, while the UK’s spans 6 April to 5 April.

The reduction in hospitalisations during the ‘lockdown year’ suggests that restricted physical activities, reduced travel, and limited social interactions significantly contributed to fewer incidents leading to oro-facial trauma, such as accidents, sports injuries, or interpersonal violence. The subsequent increase in hospitalisations during the post-lockdown period reflects a return to pre-lockdown activity levels, including increased mobility, sports participation, and social events, which elevated the risk of oro-facial injuries. However, the ASR of oro-facial hospitalisations in the post-lockdown period remained significantly lower than in the pre-lockdown period in both countries. If post-lockdown rates remain below pre-lockdown levels, it may indicate lasting changes in societal behaviour, healthcare access, or injury reporting practices influenced by the pandemic. People might have altered their lifestyles during the pandemic, and some of those changes may have become permanent. In addition, increased remote working and reduced large-scale gatherings may result in fewer opportunities for trauma-inducing interactions, such as physical altercations or commuting. Moreover, the reduction over time might be attributed to advancements in protective technology or the growing role of social media in raising awareness about personal safety. On the other hand, the pandemic may have led to a cultural shift in how minor injuries are managed. Many individuals might now prefer home remedies or primary care rather than seeking hospital treatment, which could also explain the observed trends.

While both countries experienced approximately similar lockdown-year reductions (38.8% Au, 35.7% UK), the post-lockdown decline was more sustained in Australia (35.5%) compared to the UK (25.1%). This difference may be influenced by variations in lockdown policies, healthcare responses, and societal behaviours in the two regions.

Future research is encouraged to explore trends in oro-facial hospitalisations, and assessing long-term trends in the incidence rate ratio of major to minor trauma is recommended to determine whether post-lockdown changes persist over time. A sustained decrease in trauma rates could indicate improved societal safety practices and enhanced public health measures, representing a potential positive outcome of pandemic lockdowns. Conversely, it may highlight persistent challenges in accessing care or underreporting of trauma cases. Examining these patterns is essential for guiding policy development, optimising resource allocation, and shaping preventive strategies, enabling public health systems to respond effectively to evolving circumstances.

## 5. Conclusions

The findings highlight the importance of external factors (e.g., mobility and social behaviour) in influencing oro-facial trauma rates. This insight can guide the design of preventive strategies, such as targeted interventions during periods of high risk (e.g., holidays, sports seasons, or public health crises).

In summary, the lockdown acted as a natural intervention, reducing trauma rates, while the subsequent increase reflects the resumption of regular activities, albeit with possible long-term shifts in patterns.

## Figures and Tables

**Table 1:** Demographic oro-facial trauma hospitalisations (Major and minor trauma conditions) for the pre-lockdown period (2016–19), ‘lockdown year’ (2019–20), and post-lockdown period (2020–23) among Australian adults (20+ aged).

Numbers		Pre-Lockdown Period		‘Lockdown Year’		Post-Lockdown Period	
	Age	Major	minor	Major	minor	Major	minor
Younger		5900	1123	4966	1164	5597	908
Middle		2567	949	2498	903	2335	1170
Elderly		3363	1065	3550	1183	4026	1286
All Adults		11,831	3136	11,014	3250	11,957	3365

**Table 2:** ASR oro-facial trauma hospitalisations (Major and minor trauma conditions) during the pre-lockdown period (2016–19), ‘lockdown year’ (2019–20), and post-lockdown period (2020–23) among Australian adults.

ASR * Oro-Facial Trauma Hospitalisations:			
Age Group	Mean Pre-Lockdown Period	‘Lockdown Year’	Mean Post-Lockdown Period
Younger	145.47	83.45	88.53
Middle-aged	83.91	52.81	53.70
Elderly	128.44	84.22	89.69
Adults (20+)	120.07	73.51	77.39
ASR * **Major** oro-facial trauma hospitalisations:			
Adults (20+)	94.91	56.76	60.40
ASR * **minor** oro-facial trauma hospitalisations:			
Adults (20+)	25.16	16.75	16.99

* ASR calculated per 100,000 population for each age groups.

## Data Availability

The data that support the findings of this study are openly available at the addresses listed in reference numbers [7,8,9,10].

## Abbreviations

The following abbreviations are used in this manuscript:

COVID	Coronavirus Disease
UK	United Kingdom
ICD-10	International Classification of Disease, 10th Revision
ASR	Age-Standardised Rate
AIHW	Australian Institute of Health and Welfare
NHS	National Health Service
ABS	Australian Bureau of Statistics
ONS	Office for National Statistics
CI	Confidence Interval
IRR	Incidence Rate Ratio
